# Supplementary figures and images for: Protective Effects of Centella asiatica Against Senescence and Apoptosis in Epidermal Cells
Source: Biology (Basel). 2025 Feb 14;14(2):202. doi: 10.3390/biology14020202 (PMC11851956; doi:10.3390/biology14020202)

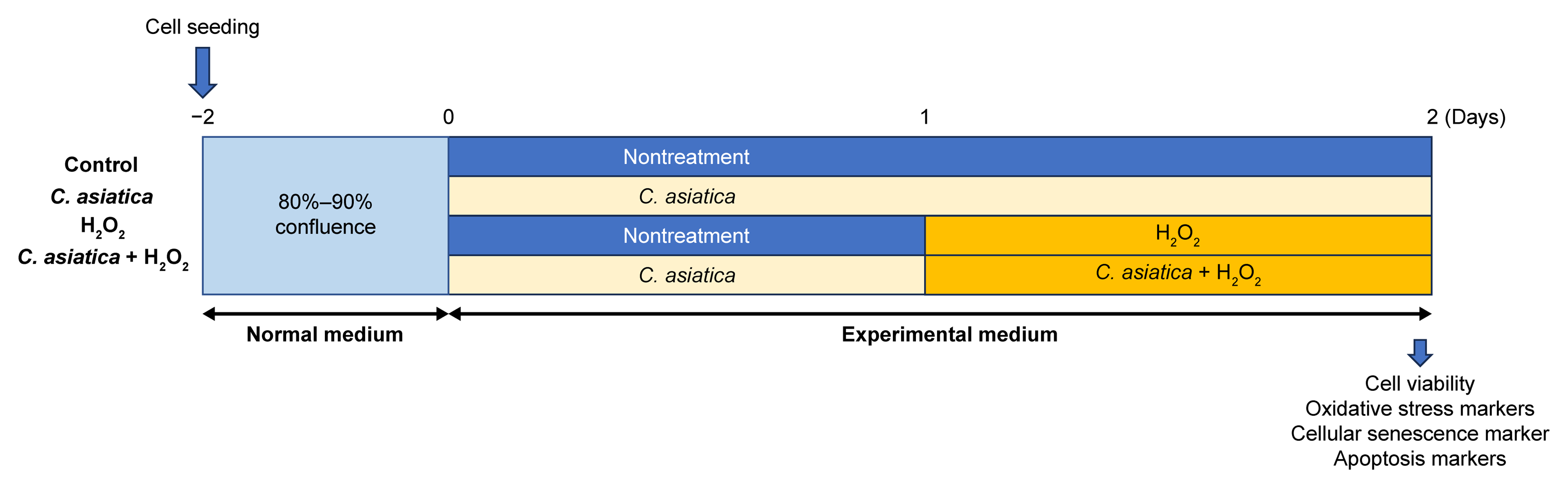

Supplement: Supplementary file 1 [file biology-14-00202-s001.zip › biology-3415678-Supplementary Figure S1.tif]
